# Effects of Desiccation and Freezing on Microbial Ionizing Radiation Survivability: Considerations for Mars Sample Return

**DOI:** 10.1089/ast.2022.0065

**Published:** 2022-10-31

**Authors:** William H. Horne, Robert P. Volpe, George Korza, Sarah DePratti, Isabel H. Conze, Igor Shuryak, Tine Grebenc, Vera Y. Matrosova, Elena K. Gaidamakova, Rok Tkavc, Ajay Sharma, Cene Gostinčar, Nina Gunde-Cimerman, Brian M. Hoffman, Peter Setlow, Michael J. Daly

**Affiliations:** ^1^ School of Medicine, Uniformed Services University of the Health Sciences (USUHS), Bethesda, Maryland, USA.; ^2^ Department of Chemistry and Life Science, United States Military Academy, West Point, New York, USA.; ^3^ Henry M. Jackson Foundation for the Advancement of Military Medicine, Bethesda, Maryland, USA.; ^4^ Department of Molecular Biology and Biophysics, UConn Health, Farmington, Connecticut, USA.; ^5^ Institute for Genetics and Cologne Excellence Cluster on Cellular Stress Responses in Aging-Associated Diseases (CECAD), Cologne, Germany.; ^6^ Center for Radiological Research, Columbia University Irving Medical Center (CUIMC), New York, New York, USA.; ^7^ Slovenian Forestry Institute, Ljubljana, Slovenia.; ^8^ Department of Chemistry, Northwestern University, Evanston, Illinois, USA.; ^9^ Biotechnical Faculty, University of Ljubljana, Ljubljana, Slovenia.; ^10^ Member, Committee on Planetary Protection (CoPP), National Academies of Sciences, Washington, DC, USA.

**Keywords:** Planetary protection, Mars, Desiccation, Freezing, Ionizing radiation, ROS, Mn antioxidants, DNA repair, Holliday junction, EPR, Vegetative cells, Spores, *Deinococcus*, *Escherichia*, *Bacillus*, *Saccharomyces.*

## Abstract

Increasingly, national space agencies are expanding their goals to include Mars exploration with sample return. To better protect Earth and its biosphere from potential extraterrestrial sources of contamination, as set forth in the Outer Space Treaty of 1967, international efforts to develop planetary protection measures strive to understand the danger of cross-contamination processes in Mars sample return missions. We aim to better understand the impact of the martian surface on microbial dormancy and survivability. Radiation resistance of microbes is a key parameter in considering survivability of microbes over geologic times on the frigid, arid surface of Mars that is bombarded by solar and galactic cosmic radiation. We tested the influence of desiccation and freezing on the ionizing radiation survival of six model microorganisms: vegetative cells of two bacteria (*Deinococcus radiodurans, Escherichia coli*) and a strain of budding yeast (*Saccharomyces cerevisiae*); and vegetative cells and endospores of three *Bacillus* bacteria (*B. subtilis, B. megaterium, B. thuringiensis*). Desiccation and freezing greatly increased radiation survival of vegetative polyploid microorganisms when applied separately, and when combined, desiccation and freezing increased radiation survival even more so. Thus, the radiation survival threshold of polyploid *D. radiodurans* cells can be extended from the already high value of 25 kGy in liquid culture to an astonishing 140 kGy when the cells are both desiccated and frozen. However, such synergistic radioprotective effects of desiccation and freezing were not observed in monogenomic or digenomic *Bacillus* cells and endospores, which are generally sterilized by 12 kGy. This difference is associated with a critical requirement for survivability under radiation, that is, repair of genome damage caused by radiation. *Deinococcus radiodurans* and *S. cerevisiae* accumulate similarly high levels of the Mn antioxidants that are required for extreme radiation resistance, as do endospores, though they greatly exceed spores in radioresistance because they contain multiple identical genome copies, which in *D. radiodurans* are joined by persistent Holliday junctions. We estimate ionizing radiation survival limits of polyploid DNA-based life-forms to be hundreds of millions of years of background radiation while buried in the martian subsurface. Our findings imply that *forward* contamination of Mars will essentially be permanent, and *backward* contamination is a possibility if life ever existed on Mars.

## Highlights

- Ionizing radiation survivability of polyploid microorganisms that accumulate Mn antioxidants (H-Mn) is greatly increased by desiccation and freezing.
- Ionizing radiation resistance of desiccated and frozen *D. radiodurans* cells far exceeds radiotolerance of *Bacillus* spores commonly used to gauge bioburden levels in *forward* planetary protection.
- Radioresistance of *D. radiodurans* relies jointly on accumulation of H-Mn antioxidants and the presence of multiple genome copies linked by Holliday junctions.
- Desiccated and frozen *D. radiodurans* survive ionizing radiation doses equivalent to hundreds of millions of years of background radiation on Mars, with implications for the possibility that dormant life endures there.
- Implications are presented regarding both *forward* and *backward* planetary contamination by Mars missions and sample return.

## 1. Introduction

Evidence for persistent liquid water on ancient Mars and significant amounts of contemporary water ice, along with the planet's proximity to Earth, have made Mars a target in the search for existing or extinct extraterrestrial life (National Academies of Sciences, Engineering, and Medicine, 2021). In addition to the growing number of national space agencies undertaking Mars exploration, private-sector enterprises are engaged in activities designed to enable manned missions. The search for evidence of past or present martian life is evolving to include sample return operations (National Aeronautics and Space Administration, 2022). To better protect Earth and its biosphere from potential extraterrestrial sources of contamination, as set forth in the Outer Space Treaty of 1967 (United Nations, 1967; Committee on Space Research, 2020), international efforts to develop planetary protection measures have strived to understand the danger of cross-contamination processes in Mars sample return missions. Toward this goal, we consider the impact of the history of the martian surface that is frozen (∼210 K, -63°C), dry, and ravaged by solar radiation and galactic cosmic radiation (GCR) on microbial dormancy and survivability.

The global scientific community has embraced the following two governing principles of planetary protection. First, spacecraft should not carry terrestrial biological materials that could compromise scientific studies of the origins or existence of life-forms on extraterrestrial bodies. This principle establishes the objective of preventing harmful *forward* contamination. Second, Earth must be protected against the introduction of microorganisms carried by spacecraft returning from another solar system body. This principle establishes the objective of preventing harmful *backward* contamination (United Nations, 1967; Committee on Space Research, 2020; National Academies of Sciences, Engineering, and Medicine, 2021).

The atmospheric pressure on Mars (∼600 Pa) is less than 1% that of Earth (∼100 kPa), and the atmosphere is so dry as to be highly desiccating (Banfield *et al.,* 2020). Life as it is understood is based on aqueous chemical reactions; liquid water is the solvent for life, and desiccation alone will prevent cell proliferation. However, under conditions of desiccation, many unicellular and even some multicellular organisms can enter a state of dormancy, in which intracellular water is lost and metabolism is undetectable, yet can resume activity upon aquation. As examples, invertebrate animals such as rotifers and some nematodes are extremely resistant to desiccation and can survive in a cryptobiotic state for thousands of years, from which they can recover upon rehydration (Krisko *et al.,* 2012; Shatilovich *et al.,* 2018; Hibshman *et al.,* 2020). Archaea, bacteria, and fungi also possess a number of strategies that allow them to survive desiccation in the form of spores or when simply dried as vegetative cells (Mattimore and Battista, 1996; Setlow, 2007; Fredrickson *et al.,* 2008; Anderson *et al.,* 2012; Gaidamakova *et al.,* 2022).

One, thus, cannot assume *a priori* that all putative martian life has become extinct. If life existed on Mars, it likely would have evolved, as the martian atmosphere dwindled, to be anaerobic and increasingly resistant to cold and dry conditions. Isolated environments such as lava tubes or caves might have provided durable protection from the planet's increasingly extreme climate (Carrier *et al.,* 2020; Sauro *et al.,* 2020). Unlike on Earth, the surface of Mars has remained mostly unchanged over billions of years, during which time its surface environments have remained desiccated and frozen (Carr and Head, 2010). However, fluctuations in the frozen martian surface environments caused by tens of thousands of meteorite impacts over its history could present the microbial population with periodic melts and habitable environments, which allow metabolic activity, repair of damage, and repopulation (Daubar *et al.,* 2013; Dundas *et al.,* 2014); the relatively recent 10 km Zunil crater is only 1–10 million years old and located in the Cerberus Plains of Mars (Preblich *et al.,* 2007).

In continuously frozen martian environments at depths greater than 10 m, the viability of dormant life would be limited only by the effects of background radiation, which is likely to be the same as on Earth (∼0.5 mGy/yr), and thus could allow a prolonged continuation of dormant life at such depths (Richmond *et al.,* 1999). The NASA Mars Sample Return (MSR) campaign is presently focused on near-surface environments that are accessible to the Perseverance rover. Notably, these surface environments are exposed to the sterilizing effects of solar ultraviolet C (UVC) (200–280 nm) radiation (non-ionizing), which reaches a maximum of 2.5–3.5 W/m^2^ (Vicente-Retortillo *et al.,* 2020), but they are also exposed to ionizing GCR and solar protons (∼76 mGy/yr) (1 Gy = 1 J/Kg = 100 rad) (Hassler *et al.,* 2014). Thus, radiation resistance is a key parameter not only in considering survival at depth, but of more immediate concern to projected sample-return missions and near-surface survivability; hence, *could radiation-resistant microorganisms reside dormant and protected within surface rock samples* (Lingappa *et al.,* 2021) *collected by rovers or simply carried by dust and wind* (Banfield *et al.,* 2020) *to sample cache containers slated for return to Earth*? For cored samples of martian rock, the dose rate for ionizing radiation within the top 10 cm of the martian subsurface is greater than, or above, that at the surface because nuclear interactions between impinging solar protons and the regolith produce short-lived isotopes that produce coincident gamma rays (∼100 mGy/yr) (Hassler *et al.,* 2014). Future missions, however, are likely to sample at depths that are more shielded from GCR and solar protons; both ExoMars (if it launches) and the Mars Life Explorer mission concept prioritized by the *Decadal Strategy for Planetary Science and Astrobiology* (National Academies of Sciences, Engineering, and Medicine, 2022) would carry drills to extract materials from 2 m below the surface (∼80 mGy/yr).

In the absence of knowledge of the existence or characteristics of life on Mars, we use microbes on Earth to estimate the ionizing radiation survival limits of DNA-based life-forms. Among the *universal* mechanisms required for these life-forms to survive the hyperoxic conditions of desiccation in Earth's atmosphere, which contains 22% dioxygen (O_2_), are antioxidant defenses, as typified by the enzyme Mn-dependent superoxide dismutase (MnSOD) (Gaidamakova *et al.,* 2022). In contrast, ionizing radiation resistance relies not on MnSOD but instead on small-molecule Mn antioxidants accumulated in the form of Mn^2+^-metabolite complexes (denoted H-Mn) (Gaidamakova *et al.,* 2022). Both antioxidants, MnSOD and H-Mn, catalyze the same reactions related to the removal of superoxide radicals (O_2_^•−^) (Gaidamakova *et al.,* 2022). However, in desiccation, O_2_^•−^ is derived mostly from atmospheric O_2_ at the cell surface and removed by MnSOD; while under ionizing radiation, O_2_^•−^ is released by decomposition of radiogenic hydrogen peroxide (H_2_O_2_) inside cells and removed by Mn antioxidants (Sharma *et al.,* 2017; Gaidamakova *et al.,* 2022).

Significantly, O_2_ is virtually absent in the thin martian atmosphere (Trainer *et al.,* 2019), so desiccation on Mars does not present the same threat as on Earth, nor does it demand the same MnSOD protection. However, because the ancient frozen environments of Mars are exposed to unremitting background radiation, H-Mn may be an essential antioxidant for martian life. In addition, irradiated water ices become enriched in H_2_O_2_, including in cells (Carlson *et al.,* 1999; Daly *et al.,* 2007). Again, H-Mn may be essential because the high iron content of such martian environments would naturally cause oxidative stress via Fenton chemistry once ice melts. The influences of desiccation and ionizing radiation on survivability are thus coupled because limiting water in cells exposed to ionizing radiation will limit the production of reactive oxygen species (ROS) generated by water radiolysis (Daly, 2009). We hypothesized that an environment that lessens the water content of cells without killing them should increase ionizing radiation survival, especially for organisms with high Mn antioxidant content and natural desiccation tolerance. In addition to antioxidant defenses, the survival of extant life would likely also require robust genomic repair systems to reassemble genomes nonetheless broken by the direct effects of background radiation over hundreds of millions of years (Richmond *et al.,* 1999). We thus further hypothesized that cells lacking sufficient genome redundancy would succumb to DNA damage irrespective of their antioxidant defenses.

These hypotheses build on the two molecular hallmarks of extremely radioresistant microorganisms as exemplified by *Deinococcus radiodurans* (Daly, 2012): the first is metabolic and mediated by the hyperaccumulation of H-Mn needed to prevent ROS damage to the proteome (Daly *et al.,* 2004, 2010); the second is genetic and mediated by the need for homologous recombination, the process responsible for maintaining genome stability in all living organisms and particularly important for repairing DNA double strand breaks (DSBs) (Daly *et al.,* 1994a, 1994b; Daly and Minton, 1995, 1996). We tested these hypotheses on six model microorganisms of differing Mn antioxidant content and differing genome copy-number, which represent the gamut of ionizing radiation and desiccation survivability: two vegetative bacteria (*D. radiodurans, Escherichia coli*), three *Bacillus* endospore forms and their vegetative counterparts (*B. subtilis, B. megaterium, B. thuringiensis*), and one budding yeast (*Saccharomyces cerevisiae*).

We report that desiccated and frozen cells of the bacterium *D. radiodurans* can survive astonishing ionizing radiation exposures of 140 kGy. For organisms buried in the subsurface below 10 m, this is equivalent to the exposure to background radiation for 280 million years. Such exposure greatly exceeds the radiotolerance and longevity determined for *Bacillus* endospores (Nicastro *et al.,* 2002), which could survive only 12 million years under similar exposure. An essential component to our considerations is provided by an updated formulation of the roles of Mn antioxidants and genome multiplicity in the ionizing radiation survivability of microorganisms.

We further address the issue of *forward* contamination. In planetary protection, bioburden constraints on spacecraft are defined with respect to the number of *Bacillus* spores that survive on their surfaces before launch. Indeed, *Bacillus* spores are renowned for their resistance to heat, desiccation, oxidative stress, solvents, UVC and ionizing radiation (Ghosh *et al.,* 2011; Granger *et al.,* 2011; Setlow, 2016). Thus, our model organisms serve as proxies for *forward* contamination as well as for *backward* contamination.

## 2. Results

We begin by showing that the dose of gamma radiation needed to sterilize a culture of 10^7^ cells (survival limit) of *D. radiodurans* can be greatly increased, from the already high value of 25 kGy in liquid culture to 140 kGy when the cells are desiccated for 5 days and then frozen and stored at -80°C. Next, we show that the radioprotective synergism between desiccation and freezing in *D. radiodurans* extends to other vegetative cell types; however, this does not extend to their spores, although they nonetheless accumulate high concentrations of Mn antioxidants (H-Mn), gauged by electron paramagnetic resonance (EPR) spectroscopy. Finally, we present the case that the multiple identical chromosomes and plasmids in *D. radiodurans* are joined by persistent 4-stranded DNA crosslinks, Holliday junctions, which are predicted to facilitate DNA repair.

### 2.1. Ionizing radiation survival limits of desiccated *D. radiodurans*

The phenotypes of desiccation resistance and extreme ionizing radiation resistance are coupled across the tree of life, with the most resistant representatives accumulating both MnSOD enzymes and Mn antioxidants, as exemplified by bacteria of the genus *Deinococcus* (Gaidamakova *et al.,* 2022). In liquid culture, *D. radiodurans* (ATCC BAA-816) can endure (37% survival) acute exposures to gamma radiation of 17.5 kGy that cause ∼200 DSBs per haploid genome (3.3 Mbp). As the cells maintain 4–8 identical copies of its chromosomes and plasmids during growth and stationary-phase, this corresponds to 800–1600 DSB fragments per cell (Daly *et al.,* 1994a, 1994b; Daly and Minton, 1995, 1996).

**FIG. 1.**
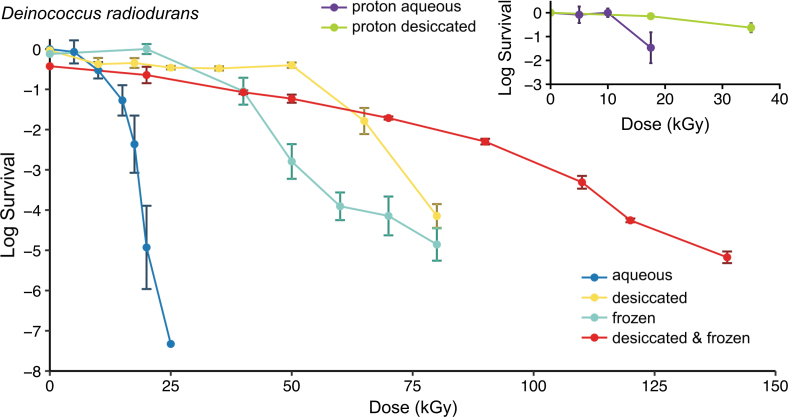
Gamma radiation resistance of *D. radiodurans* (ATCC BAA-816). Following irradiation, cell survival was monitored by CFU assay. Note, the “survival-limit” is the lowest radiation dose that eliminates all CFUs; aqueous, on wet ice (0°C); frozen, on dry ice (-79°C); desiccated (5 days). (Inset) Proton irradiations were at room temperature and terminated at 35 kGy, which are operational parameters set by the Radiological Research Accelerator Facility (RARAF) and do not represent the survival limit of desiccated *D. radiodurans* cells for proton irradiation. Data normalization: logarithmic survival values of a culture subjected to desiccation and/or freezing conditions compared to the original aqueous culture of non-irradiated cells (∼1 × 10^8^ CFU/mL). Results and SD error bars represent data from three biological replicates.

The *D. radiodurans* gamma radiation survival curves of Fig. 1 are for cells irradiated in aqueous, frozen, or desiccated states, and each is built from three biological replicates grown and recovered under nutrient-replete conditions (tryptone-glucose-yeast extract [TGY] medium), as described previously (Daly *et al.,* 2004; Gaidamakova *et al.,* 2022). The inset of Fig. 1 is for proton irradiation to simulate solar ionizing radiation. Specifically, *D. radiodurans* cells were grown to the early stationary phase when they are the most resistant (Daly *et al.,* 1994a; Gaidamakova *et al.,* 2022). Cells were transferred to 96-well polystyrene microtiter plates and transferred to chambers containing Drierite desiccant (calcium sulfate) (Fredrickson *et al.,* 2008; Gaidamakova *et al.,* 2022). Our data show that desiccating the bacterium *D. radiodurans* for 5 days before irradiation greatly extended its radiation survival and extended survival even further when the cells were frozen after desiccation (Fig. 1).

### 2.2. Radioprotective synergism between desiccation and freezing is observed in other vegetative cell types

**FIG. 2.**
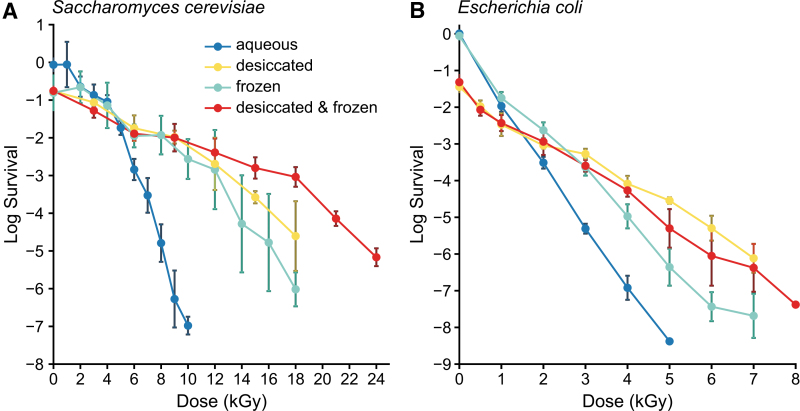
Gamma radiation resistance of *S. cerevisiae* (EXF-6761) (**A**) and *E. coli* (MG1655) (**B**). Following irradiation, cell survival was monitored by CFU assay, with SD error bars representing data from three biological replicates. Data normalization as in Fig. 1.

We determined the gamma radiation survival limits of *S. cerevisiae* (EXF-6761) and *E. coli* (MG1655) grown to the early stationary phase then desiccated (5 days) and frozen (-79°C). The yeast *S. cerevisiae* (EXF-6761) is a highly radioresistant diploid strain that displays radioprotective synergism between desiccation and freezing that is greater than for the radiosensitive bacterium *E. coli* (Sharma *et al.,* 2017). The survival limit of *S. cerevisiae* (EXF-6761) increased from 10 kGy in liquid medium (0°C) to 24 kGy when desiccated and frozen (Fig. 2A), whereas the survival-limit of *E. coli* increased from 5 kGy in liquid medium to 8 kGy when the cells were desiccated and frozen (Fig. 2B).

### 2.3. Freezing and desiccation does not increase the radioresistance of *Bacillus* endospores that nonetheless hyperaccumulate Mn antioxidants, gauged by EPR

**FIG. 3.**
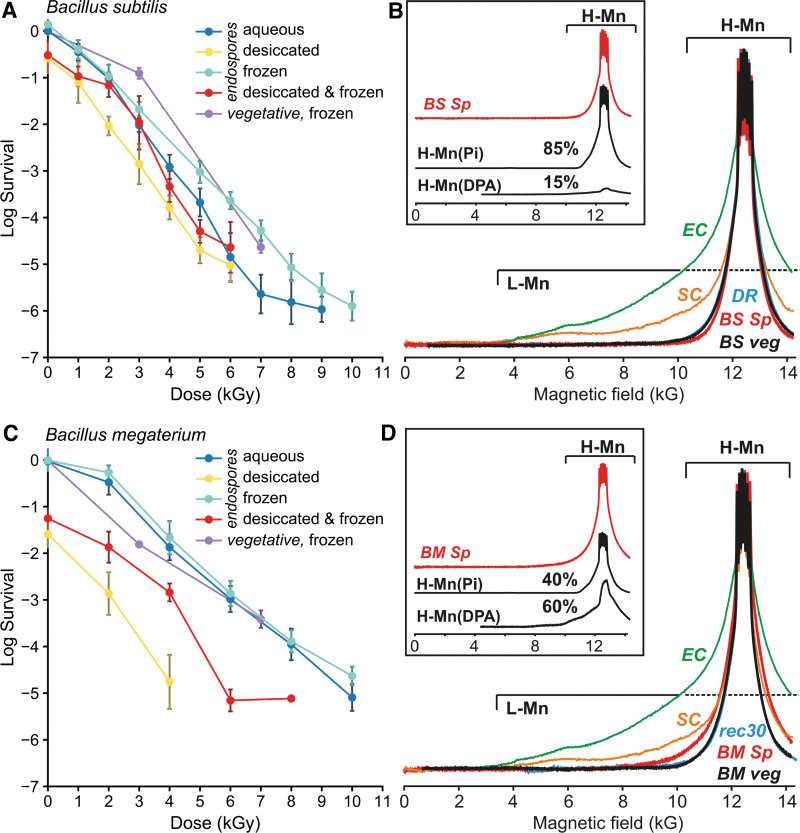
Gamma radiation resistance and H-Mn content of *B. subtilis* and *B. megaterium*. (**A**) Survival curves for *B. subtilis* (PS533) spores and vegetative cells. Following irradiation, cell survival was monitored by CFU assay, with SD error bars representing data from three experimental replicates. Data normalization as in Fig. 1. (**B**) Absorption display 35 GHz continuous-wave (CW) EPR spectra for *B. subtilis* spores (*BS Sp,* red) and vegetative cells (*BS veg,* black). These spectra, like those of *D. radiodurans* (wild-type) (*DR,* cyan), are dominated by narrow signals from H-Mn antioxidants, in contrast to the spectra of *E. coli* (MG1655) (*EC,* green), and *S. cerevisiae* (EXF-6761) (*SC,* orange), which include substantial contributions from the broad L-Mn component signal, which exhibits “wings” down to under 4 kG (see text). To highlight the difference in the appearance of spectra with and without large contributions from L-Mn, the EPR spectra are normalized to the same maximum amplitude. (Inset) Decomposition of the H-Mn EPR spectrum of *B. subtilis* spores (*BS Sp*) into fractional contributions (shown as %) from spectra of the two reference exemplars, H-Mn(Pi) and H-Mn(DPA) (See Fig. S3). However, as noted in the text, the spore H-Mn(Pi) component of the spectrum includes contributions not only from Mn complexed with inorganic phosphate (Pi) but also from the similar spectra of Mn complexed with the large amounts of 3-phosphoglyceric acid found in spores of all *Bacillus* species examined, in some cases with levels even higher than Pi (Camilleri *et al.,* 2019; Ghosh *et al.,* 2011). (**C**) Survival of *B. megaterium* (QMB1551) spores and vegetative cells irradiated as in (A). Following irradiation, cell survival was monitored by CFU assay, with SD error bars representing data from three experimental replicates. Data normalization as in Fig. 1. (**D**) H-Mn CW-EPR spectra for *B. megaterium* spores (*BM Sp,* red) and vegetative cells (*BM veg,* black) compared to *D. radiodurans* (*rec30, recA*^-^) (*rec30,* cyan), again with the contrasting spectra from *E. coli* (MG1655) (*EC,* green, as in panel B), and *S. cerevisiae* (EXF-6761) (*SC,* orange). Again, to highlight the difference in the appearance of spectra with and without large contributions from L-Mn, the EPR spectra are normalized to the same maximum amplitude. (Inset) Decomposition of H-Mn EPR spectra of *B. megaterium* spores (*BM Sp*) again gives percentage contributions to H-Mn spectra from the two exemplar spectra of H-Mn(Pi) and H-Mn(DPA) (see also Fig. S2). See also Fig. S1, *B. thuringiensis* survival and EPR. Abbreviations: Pi = inorganic phosphate; DPA = dipicolinic acid.

We previously reported that gamma radiation survival of *Bacillus* spores in liquid is independent of their Mn levels, which nonetheless are important factors in determining resistance to other environmental stressors such as heat and H_2_O_2_ (Ghosh *et al.,* 2011; Granger *et al.,* 2011; Paruthiyil *et al.,* 2020). Furthermore, the survival limit of *Bacillus* spores is approximately 10 kGy regardless of their physiochemical state: as liquid suspensions held on wet ice (0°C); as aqueous frozen suspensions held on dry ice (-79°C); as desiccated spores irradiated at 0°C; as desiccated spores irradiated at -79°C; or as frozen vegetative cells grown in Luria-Bertani (LB) medium to the early stationary phase (Fig. 3A and 3C; see also Fig. S1A for *B. thuringiensis*).

*Bacillus* spores and vegetative cells accumulate Mn^2+^ (Ghosh *et al.,* 2011; Granger *et al.,* 2011; Paruthiyil *et al.,* 2020), but the level of Mn antioxidants (H-Mn) that actually form in spores had not been measured to date. Standard analytical biochemical procedures cannot determine whether intracellular H-Mn is present in spores, and if it is, what types of complexes there are. Breaking open spores alters the chemical speciation of Mn^2+^ and destroys information about the *in vivo* Mn speciation. Instead, we employed EPR spectroscopy to identify cellular Mn-speciation and measure Mn antioxidant content in intact *Bacillus* spores and vegetative cells (Sharma *et al.,* 2017; Gaidamakova *et al.,* 2022).

The 35 GHz continuous-wave (CW) absorption-display Q-band EPR spectra of cellular Mn have contributions from two Mn pools. The first pool is the H-Mn antioxidants, which display a relatively narrow signal, whose peak exhibits a sharp sextet created by electron-nuclear hyperfine interactions with the ^55^Mn nucleus (Fig. 3B and 3D; see also Fig. S1B). The spectrum of this pool has been modeled previously as a weighted sum of two component spectra. One is the spectrum of phosphate-bound Mn^2+^ (denoted as Mn-Pi or H-Mn(Pi)), which is indistinguishable from the spectrum of Mn^2+^(H_2_O)_6_, as well as from the spectra of a variety of other phosphate and carboxylate complexes, including 3-phosphoglycerate found at high levels in spores (Granger *et al.,* 2011; Paruthiyil *et al.,* 2020); electron-nuclear double resonance (ENDOR) spectroscopy can aid in distinguishing among these (McNaughton *et al.,* 2010). The second component is the somewhat broader spectrum that previously was modeled with the spectrum of imidazole-bound Mn^2+^ (Mn-Imi or H-Mn(Imi)) (Sharma *et al.,* 2017). We now find that the spectra of Mn-Imi and Mn-dipicolinic acid (Mn-DPA) are equivalent for this purpose (Fig. S2, left). Taking into consideration the high abundance of dipicolinic acid (DPA) in spores, but *not* in vegetative cells (Granger *et al.,* 2011; Camilleri *et al.,* 2019), we treated, for consistency, the H-Mn signal in the spores as a weighted sum of Mn-Pi and Mn-DPA spectra, but in the vegetative cells as a sum of Mn-Pi and Mn-Imi spectra, while emphasizing that reversal of the procedure showed that the choice has no influence on any conclusions (see Fig. S3).

In addition to the H-Mn pool, there exists the second cellular Mn-pool, “L-Mn,” complexes, whose signals are quite distinct from those of H-Mn, namely, from H-Mn(Pi), H-Mn(Imi), and H-Mn(DPA), being characterized by broad “wings” extending from low fields, ∼3 kG, to high fields well beyond the magnet limit (Fig. 3B and 3D; see also Figs. S1B and S2). The L-Mn signal is associated with a heterogeneous population of low-symmetry Mn^2+^-bound complexes and enzymes that do not afford antioxidant radioprotection (Sharma *et al.,* 2017; Gaidamakova *et al.,* 2022).

Partitioning the EPR spectra of *Bacillus* spores and vegetative cells into the several exemplar contributions (Fig. 3B and 3D; see also Fig. S1B for *B. thuringiensis*) shows that all contain negligible amounts of L-Mn, instead hyperaccumulating the H-Mn antioxidant species at amounts equivalent to those of the most ionizing radiation–resistant organisms known, including *D. radiodurans* bacteria (Daly, 2012; Sharma *et al.,* 2017), *Chroococcidiopsis* cyanobacteria (Billi *et al.,* 2000), and *Caenorhabditis elegans* nematodes (Gaidamakova *et al.,* 2022). These characteristics are in contrast to those of the archetypal radiation-sensitive *E. coli*, for example, which instead contains a large pool of non-radioprotective L-Mn and only a small pool of H-Mn antioxidants (Fig. S2, right) (Sharma *et al.,* 2017).

Distinctively, the EPR partitioning shows that, while an EPR contribution from the Mn-DPA exemplar spectrum is absent from *Bacillus* vegetative cells, it is a major component of the dominant H-Mn pool in endospores. The reason for this difference is that vegetative cells have high levels of a variety of metabolic intermediates that can bind Mn, producing H-Mn. The absence of a contribution of the component Mn-DPA exemplar spectrum to the spectrum of the H-Mn pool in *Bacillus* vegetative cells implies that the formation of the H-Mn in these cells primarily involves small-molecule metabolites other than DPA, including phosphorylated metabolic intermediates and protease-derived peptides, as occurs in *D. radiodurans* (Daly *et al.,* 2010). Almost all these metabolic intermediates are absent in dormant spores, and the EPR partitioning suggests that Mn-DPA may be a major component of the H-Mn of the digenomic spores of *B. megaterium,* and *B. thuringiensis,* but less so in the monogenomic *B. subtilis* spores (Fig. 3B and 3D; see also Fig. S1B).

### 2.4. Role of genome multiplicity and Holliday junctions in *D. radiodurans* survival

The H-Mn contents of *D. radiodurans* and of *Bacillus* spp. vegetative cells and spores are *all* exceptionally high; thus the cells and spores would be expected to be similarly resistant to gamma radiation (Ghosh *et al.,* 2015; Sharma *et al.,* 2017; Gaidamakova *et al.,* 2022). Yet the *Bacillus* spores are in fact much less resistant than *D. radiodurans* and *S. cerevisiae* (EXF-6761) (compare Figs. 1, 2A, 3A, and 3C; see also Fig. S1A). The simplest explanation for this apparent contradiction is genomic redundancy (*i.e.,* genome copy-number), which naturally governs the level of homologous DSB repair required for survival in irradiated cells (Daly *et al.,* 1994a, 1994b; Daly and Minton, 1995, 1996). Consider first *Bacillus* endospores, which are either exclusively monogenomic (*e.g., B. subtilis*) or digenomic (*e.g., B. megaterium* and *B. thuringiensis*) (Hauser and Karamata, 1992), as is the case for many vegetative bacteria including *E. coli,* which contain 1–2 genome copies per cell. In contrast, the diploid yeast *S. cerevisiae,* which in G2 is tetraploid, carries 2–4 genome copies per cell as two sets of sister chromatids held together by centromeres. Finally, *D. radiodurans* bacteria are polyploid and maintain as many as 8 genome copies per cell throughout the growth cycle (Slade and Radman, 2011; Ohbayashi *et al.,* 2019).

In considering why *D. radiodurans* is so much more resistant to radiation-induced DSBs than other organisms, we revisit a DNA repair model of Minton and Daly (1995). They hypothesized that 4-stranded DNA junctions (Holliday junctions) persistently join the multiple identical chromosomes in *D. radiodurans,* thereby *greatly* facilitating the “search for homology” during homologous recombination (Minton and Daly, 1995). The Holliday junction is a cross-shaped structure that physically links two homologous DNA helices, a universal intermediate in homologous recombination and DSB repair. The DNA repair model was built on extensive molecular genetic findings that hundreds of gamma radiation–induced DSBs in *D. radiodurans* are repaired by hundreds of DNA crossovers (Daly *et al.,* 1994a, 1994b; Daly and Minton, 1995, 1996). Of course, crossovers are derivatives of Holliday junctions, which naturally migrate across identical DNA duplexes, but such 4-stranded DNA junctions are lost off the ends of DSBs, particularly when DNA is purified (Lin *et al.,* 1999).

**FIG. 4.**
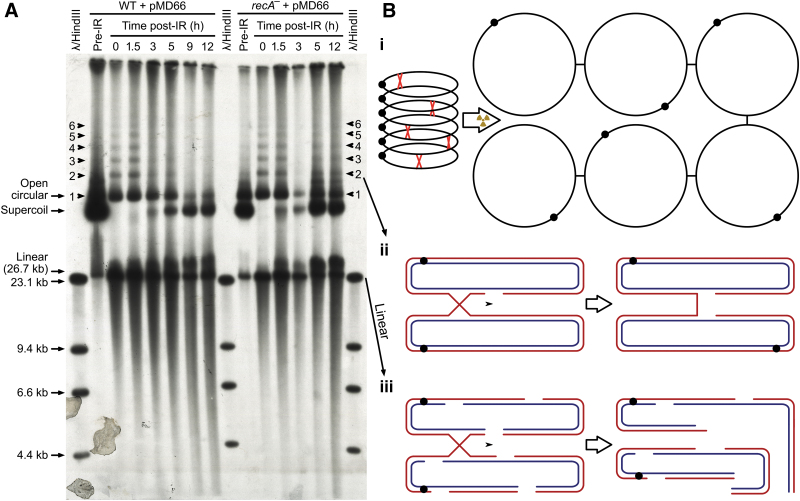
Concatemers of *D. radiodurans* plasmid pMD66 induced by gamma radiation. (**A**) Breakage and regeneration of plasmid in irradiated *D. radiodurans* wild-type R1/pMD66 and *recA^-^*/pMD66. Early stationary phase cells were gamma-irradiated with 10 kGy while on ice, and total DNA was purified immediately following, or the cells were diluted 1/50 in fresh TGY broth and allowed to incubate at 32°C with aeration for the indicated time prior to purification of DNA. Each lane contains DNA from 3 × 10^6^ cells, as determined by hemocytometer count. Electrophoresis was in a 0.55% agarose gel for 12 h at 75 V prior to blotting and probing of the blot with a unique 1.2 kb fragment of pMD66 (Fig. S4) that had been radiolabeled with (^32^P α-dCTP) by the random priming method. The markers are lambda phage DNA digested with *Hind*III. An *in vivo* dose of 10 kGy is predicted to inflict 8 SSBs and less than 1 DSB in the 26.7 kbp pMD66 plasmid; this converts SC forms into OC forms, which are then converted back to SC forms during recovery in the wild-type (WT) and *recA^-^* mutant (*rec30*) (Daly *et al.,* 1994a). *Deinococcus radiodurans* does not catalyze non-homologous DNA end-joining (NHEJ) (Daly *et al.,* 1994a, 1994b). Note that, in the 3 h *rec30,* there was sample loss upon loading the gel. (**B**) Dynamic pMD66 plasmids are joined by Holliday junctions. When envisioning supercoiled (SC), open-circular (OC), and linear plasmid forms, consider (i) 6 undamaged SC forms linked by 5 Holliday junctions (red crosses), which migrate across identical DNA duplexes; (ii) upon introduction of a few gamma radiation–induced SSBs, 6 OC cross-linked forms are generated, as detailed for an OC dimer with 1 SSB and 1 Holliday junction; and (iii) however, too many DSBs, as represented by an OC pMD66 dimer with 2 DSBs and 1 Holliday junction, release linear fragments. Of course, Holliday junctions will be lost over time due to resolvase(s), DNA repair or annihilation when two like Holliday junctions meet (Minton and Daly, 1995).

The presence of persistent Holliday junctions linking genomic copies in *D. radiodurans* was previously tested by transmission electron microscopy (TEM), but this effort failed to show interstrand crosslinks (Lin *et al.,* 1999). To reevaluate the hypothesis and TEM study, we first noted that target theory holds that DNA damage in irradiated cells is directly related to genome size (bp) and that the incidence of DSBs increases linearly with dose (Daly *et al.,* 1994a; Daly, 2012). As the TEM study applied 17.5 kGy, a dose that inflicted 3.3 DSB/kGy/Mbp in chromosomes and plasmids of *D. radiodurans* in aqueous conditions (Daly *et al.,* 1994a, 1994b; Daly and Minton, 1995; Lin *et al.,* 1999; Sharma *et al.,* 2017), we exposed cells in the present study to just 10 kGy to prevent DSBs in small plasmids and thereby preserve Holliday junctions connecting covalently closed circular (ccc) plasmids. Using the 26.7 kbp *D. radiodurans* plasmid pMD66 (Fig. S4) as a reporter of DNA conformation after irradiation (Daly *et al.,* 1994a), cells containing pMD66 were exposed to 10 kGy, a dose predicted to inflict less than 1 DSB per pMD66 molecule (3.3 × 10.0 kGy × 0.0267 Mbp). This irradiation regimen coupled to agarose gel electrophoresis enabled us to distinguish between the three structural forms of pMD66 *in vivo:* the undamaged supercoiled (SC) form, the open circular (OC) form that contains only single strand breaks (SSBs), and the linear form that results from one DSB (Daly *et al.,* 1994a). Figure 4 shows concatemers of OC DNAs, from 1-mers to 6-mers of the ccc pMD66 molecules in irradiated *D. radiodurans,* as predicted by Minton and Daly (Fig. 4A and 4B) (Minton and Daly, 1995).

## 3. Discussion

Scientists and philosophers have long pondered the origin, prevalence, and nature of life in the universe. This timeless quest is now driven by growing evidence for persistent liquid water on ancient Mars at a time that overlaps with the epoch on Earth that engendered 3.7-billion-year-old fossilized stromatolites (Allwood *et al.,* 2006). Did life also originate during that period on Mars? If so, could life-forms remain viable in the frigid, arid martian subsurface, which has been subject to unremitting background ionizing radiation over eons? The last evidence of flowing water on the surface of Mars is as recently as 2 billion years ago (Leask and Ehlman, 2022), which provides the geologic timeframe over which we must consider survival in the frozen subsurface.

The critical constraint on the possible presence of extant dormant life on Mars is prolonged exposure to ionizing radiation, though the flux depends on location. Mars lacks a magnetosphere, and the background dose rates for ionizing radiation at the surface, which, in the martian environment, would largely have been due to GCR and solar protons penetrating through the thin martian atmosphere, are considerably greater than that at Earth's surface and range from 76–96 mGy/yr (Hassler *et al.,* 2014). However, if an organism were buried 10 m below the surface of Mars, it would face only the internal planetary background radiation, mainly ionizing forms as on Earth (∼0.5 mGy/yr). Our study extends insights on the roles of Mn antioxidants and genome multiplicity in determining the possibility that dormant microorganisms have persisted on Mars over geologic times.

In reaching for answers to these questions, and in the absence of knowledge of the existence or characteristics of life on Mars, we use microbes on Earth to estimate the ionizing radiation survival limits of DNA-based life-forms and thereby help to better understand the process of cross-contamination of forward and return Mars missions. We build on the results of two prior investigators. John Battista first reported the highly radioprotective effects of drying on *D. radiodurans* (National Academies of Sciences, Engineering, and Medicine, 2002); desiccation removes water and should lower ROS levels in irradiated cells (Mattimore and Battista, 1996). Robert Richmond first reported the highly radioprotective effects of freezing *D. radiodurans* (Richmond *et al.,* 1999); freeze-concentration of solutes at ice/solution interfaces should lower ROS levels in irradiated cells by further concentrating H-Mn (Butler, 2002). In the present study, we have extended those early investigations by showing synergism between desiccation and freezing on the ionizing radiation survival of six model microorganisms and the key role of genomic redundancy.

### 3.1. Effects of desiccation, freezing, and polyploidy on radioresistance

The exceptional augmentation of ionizing radiation resistance in *D. radiodurans* upon desiccation and freezing is unmatched by the other microorganisms, including *Bacillus* endospores, that are renowned for their desiccation survival over at least many hundreds of years (Kennedy *et al.,* 1994) and resistance to ionizing radiation (Setlow, 2007; Ghosh *et al.*, 2011; Granger *et al.*, 2011; Setlow, 2016). Our previous survey of microbial Mn antioxidant content, as determined by EPR, demonstrated that the most radiation-resistant prokaryotes and eukaryotes *consistently* accumulate H-Mn, but radiosensitive microorganisms do not (Sharma *et al.,* 2017; Gaidamakova *et al.,* 2022). However, *Bacillus* spores, which hyperaccumulate Mn antioxidants, respond very differently to ionizing radiation than other H-Mn-accumulating microorganisms, depending on their physiochemical environment. Whereas radiation survivability of *Bacillus* spores and their vegetative forms remains between 4 and 11 kGy at most (Fig. 3A and 3C; see also Fig. S2A), the gamma radiation survival limit in *D. radiodurans* is extended from 25 kGy in liquid culture to 140 kGy when the cells are both desiccated and frozen (Fig. 1), and similarly for *S. cerevisiae* (EXF-6761), from 10 kGy in liquid culture to 24 kGy when desiccated and frozen (Fig. 2A).

**FIG. 5.**
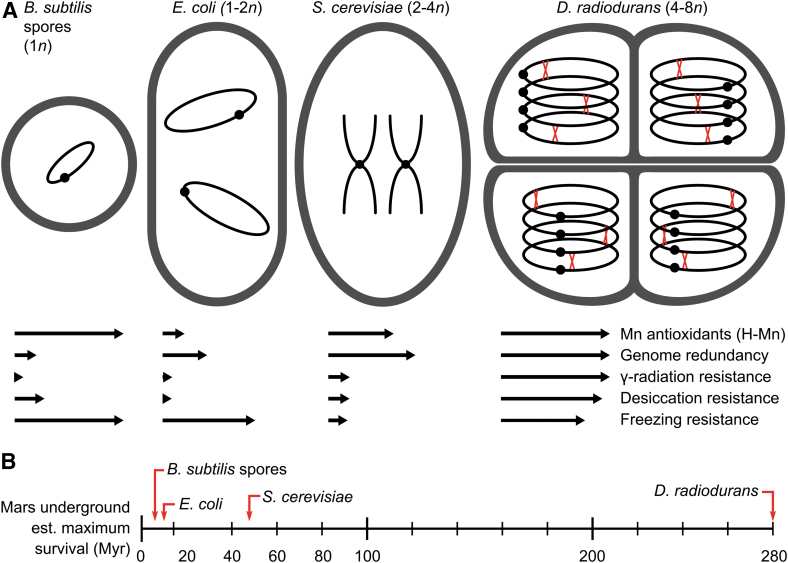
Impact of ploidy (*n*) and Mn antioxidants on survivability. (**A**) Effects of H-Mn content, desiccation, and freezing on ionizing radiation survivability of microbes with increasing genome multiplicity. Left to right, increasing ploidy: *B. subtilis* monogenomic spore; *E. coli,* 2 sets of ccc chromosomes per cell; *S. cerevisiae,* diploid, 4 linear genome copies per cell; *D. radiodurans* tetracoccus, 4–8 ccc genome copies per cell linked together by Holliday junctions (red crosses) (see Fig. 4) (Minton and Daly, 1995). Relative resistance to ionizing radiation, desiccation, and freezing is indicated by arrow length based on metrics from Figs. 1–3. (**B**) Estimated survival times of model microorganisms in the frigid and arid martian subsurface (Myr, 10^6^ years at 0.5 mGy/year internal planetary background radiation). The metrics represented here are used to qualitatively illustrate the extent of survivability as seen in DNA-based Earth organisms rather than a quantitative prediction for continuing life on Mars.

The simplest explanation for the fixed radiation survival in *Bacillus* spores is the dearth of genomic redundancy (*i.e.,* genome copy-number). Polyploidy—the existence of multiple (*n*) copies of a chromosome or set of chromosomes—is widely distributed among eukaryotes, but there are also several well-documented polyploid prokaryotic lineages, notably *Deinococcus* and *Chroococcidiopsis* (Slade and Radman, 2011; Ohbayashi *et al.,* 2019). Thus, our data show that, among microorganisms that accumulate Mn-antioxidants, ionizing radiation fitness can be greatly enhanced by either desiccation or freezing in some, but not others, depending on the presence of polyploidy. Our data also indicate that the magnitude in the increase in radiation resistance scales with genome copy-number: *D. radiodurans* (4–8*n*) > *S. cerevisiae* (EXF-6761) (2–4*n*) > *Bacillus* spp. spores (1–2*n*) (Fig. 5A) (Minton and Daly, 1995).

The multiple identical genomes in *D. radiodurans* are condensed into nucleoids, wherein the chromosomes and plasmids are highly recombinogenic (Daly *et al.,* 1994a, 1994b, 2004; Daly and Minton, 1995, 1996; Minton and Daly, 1995). We attribute the extraordinary radiation survival of *D. radiodurans* to the linkage of its genome copies, as first proposed by Minton and Daly and confirmed here (Fig. 4) (Minton and Daly, 1995). In short, if one extrapolates the findings on linkage between the *D. radiodurans* pMD66 plasmids (0.0267 Mbp) to the whole genome (3.3 Mbp), then the “search for homology” needed for the repair of hundreds of DSBs in highly irradiated *D. radiodurans* is much simplified. Such 4-stranded DNA links are resolved as genetic crossovers during DSB repair in *D. radiodurans* (Daly *et al.,* 1994a, 1994b; Daly and Minton, 1995, 1996), as is observed in yeast chromosomes undergoing repair of hundreds of DSBs during meiosis (Allers and Lichten, 2001).

Our findings thus imply that the astonishing gamma radiation survivability of *D. radiodurans* is a consequence of the coupling of two distinct mechanisms. First, the hyperaccumulation of Mn antioxidants, which preserve the functionality of the proteins under immense oxidative stress (Daly *et al.,* 2007; Daly, 2012; Sharma *et al.,* 2017), and second, the preexisting structural linkage of its 4–8 genome copies, which explains how the cells rapidly repair hundreds of DSBs caused by megadoses of gamma rays (Fig. 1) (Daly *et al.,* 1994a, 1994b; Daly and Minton, 1995, 1996; Minton and Daly, 1995). This combination results in the extraordinary radioresistance of *D. radiodurans,* but similar solutions may have evolved independently in other species. It has not escaped our notice that the aquatic and photosynthetic *Chroococcidiopsis* also hyperaccumulates H-Mn and is polyploid (Ohbayashi *et al.,* 2019); the tetracoccal cyanobacterium is extremely desiccation-resistant and can survive hundreds of gamma-radiation-induced DSBs (Billi *et al.,* 2000; Lingappa *et al.,* 2021).

### 3.2. Implications for extant life on Mars

In the absence of knowledge on the existence or characteristics of life on Mars, the present study of Earth microbes provides an estimate of the ionizing radiation survival limits of DNA-based life-forms. Starting with *D. radiodurans*, we show that desiccation and freezing increases the radiation survival limit from 25 kGy in liquid culture to 140 kGy when the dried cells are held at temperatures that approximate the surface of Mars and are then gamma-irradiated (Fig. 1). We then show that this synergism extends to some of the other vegetative microorganisms, the budding yeast *S. cerevisiae* (EXF-6761) (Fig. 2A), but less so, the bacterium *E. coli* (Fig. 2B), and not to *B. subtilis, B. megaterium,* and *B. thuringiensis* endospores (Fig. 3A and 3C; see also Fig. S1A).

As noted above, the dose rates for ionizing radiation impinging the surface of Mars are considerably greater than those on Earth's surface and range from 76 to 96 mGy/yr (Hassler *et al.,* 2014). Importantly, *D. radiodurans* is as resistant to proton irradiation as gamma irradiation (Fig. 1); therefore, the theoretical maximal survival dose of desiccated and frozen *D. radiodurans* in martian near-surface environments that are shielded from UVC should be reached in only ∼1.5 Myr. It follows that viability of *S. cerevisiae* (EXF-6761) would be extinguished in the near-surface of Mars within ∼0.25 million years (24 kGy/96 mGy/yr), and less so for frozen and desiccated *E. coli* (∼0.08 Myr) and *B. subtilis* (∼0.063 Myr) spores.

However, the internal martian planetary background radiation, mainly ionizing forms as on Earth, is instead only ∼0.5 mGy/yr. If *D. radiodurans* were buried desiccated and frozen 10 m below the surface of Mars, then the theoretical accumulated dose for overwhelming a population might be approached in 280 million years (140 kGy/0.5 mGy/yr), as visualized in the scheme of Fig. 5B. Radioprotective synergism between desiccation and freezing was also observed in *S. cerevisiae* (EXF-6761), which, like *D. radiodurans,* also expresses MnSOD and accumulates Mn antioxidants (Daly *et al.,* 2004; Sharma *et al.,* 2017) and is therefore resistant to desiccation and ionizing radiation (Gaidamakova *et al.,* 2022). Therefore, estimated microbial survival times in the martian subsurface (>10 m) are substantially greater than at the surface; *S. cerevisiae* (EXF-6761) would be expected to survive ∼48 million years (24 kGy/0.5 mGy/yr), and survival would be less so for frozen and desiccated *E. coli* (∼16 Myr) and *B. subtilis* spores (∼12 Myr) (Fig. 5B).

We have thus shown that desiccated and frozen terrestrial microorganisms can survive ionizing radiation doses equivalent to hundreds of millions of years of background radiation on Mars. It must also be noted that planetary protection scenarios similarly encompass viruses, which are the most abundant life-forms on Earth and likely evolved alongside any martian life. DNA viruses are typically much more radiation-resistant than their hosts because of the small target size of their small genomes (Daly, 2012). The question of whether transferred viruses constitute a harmful threat to Earth, and *vice versa* for Mars, depends on how closely any extant martian virus is related to life on Earth, and neither is considered likely because of virus-host incompatibility. The ability of cellular martian life to survive and proliferate if transferred to Earth is considered equally unlikely, but for a different reason: the severe oxidative stress imposed by Earth's oxygenated atmosphere would surely rule out most terrestrial habitats.

Since the Viking missions of the 1970s, planetary protection bioburden constraints on outbound Mars spacecraft have been defined with respect to the number of bacterial spores that survive on their surfaces before launch. This is accomplished by using a selection regimen that specifically targets *Bacillus* endospores, important indicator species for *forward* contamination; contaminants collected on swabs are heat-shocked at 80°C for 15 min followed by culture on trypticase soy agar at 32°C for 72 h under aerobic conditions; for NASA's current Mars planetary protection requirements, see NPR 8020_012D Sec 5.3 (NAS, 2021: the NAS Report, Bioburdon Requirements for Mars Missions.). In retrospect, despite the relatively low ionizing radiation survival limit of *Bacillus* spp. compared to *D. radiodurans* and *S. cerevisiae* (EXF-6761), spores are a good choice of sentinel organisms for contamination because the current approach for sterilizing whole spacecraft is heat, not radiation; indeed, *D. radiodurans* is readily killed by heat-shock at 80°C for 15 min. However, in consideration of *backward* contamination, the ionizing radiation survival metrics of *D. radiodurans* and *S. cerevisiae* (EXF-6761) clearly make them the references of choice.

### 3.3. Conclusions

The least frequent and most dangerous form of DNA damage caused by planetary background radiation is the double strand break (DSB). As illustrated here, the best way to enhance radiation survivability is to combine protection of repair proteins against ionizing radiation–generated ROS by high-symmetry Mn^2+^ antioxidants (H-Mn) with increased genomic redundancy that facilitates DSB repair. This paradigm is exemplified by *D. radiodurans* cells, which hyperaccumulate H-Mn and maintain 4–8 structurally linked genome copies per cell (Fig. 4) (Minton and Daly, 1995) and thereby are enabled to survive 140 kGy when desiccated and frozen (Fig. 1). With regard to planetary protection, our results support three findings: first, it must be assumed that any *forward* contamination of the martian subsurface with terrestrial microorganisms would essentially be permanent, over mission time frames of 1000s of years, and this could complicate scientific efforts to look for martian life, even though terrestrial microbes would not proliferate under martian conditions.

Second, regarding *backward* contamination, the present assessment that microorganisms could possibly survive over geologic timescales if frozen, desiccated, and buried in the subsurface means that it would be inappropriate to think that microbes that evolved on Mars would not be capable of surviving until the present day. Although even *D. radiodurans* buried in the martian subsurface could not survive dormant for the estimated 2–2.5 billion years since flowing water disappeared, such martian environments are regularly altered and melted by meteorite impacts (impact gardening), and we suggest that periodic melting could allow intermittent repopulation and dispersal. Of course, the success of such repopulation and, thus, the probability of microbes surviving exposure to eons of background radiation on Mars is inextricably linked to the availability of carbon and energy sources needed to fuel genomic and metabolic restoration (Venkateswaran *et al.,* 2000; Ghosal *et al.,* 2005).

Third, if martian life ever existed, even if viable life-forms are not now present on Mars, given that whole viable *D. radiodurans* cells can survive the equivalent of 280 million years in the frozen martian subsurface, then their macromolecules would survive much, much longer. This strengthens the probability that, if life ever evolved on Mars, this will be revealed in future missions.

## 4. Materials and Methods

### 4.1. Cell growth and gamma irradiation

*Deinococcus radiodurans* strains were WT strain R1 (ATCC BAA-816) and strain *rec30* (*recA*^-^) (Daly *et al.,* 1994a, 1994b). Bacteria were inoculated into defined liquid TGY medium (1% bactotryptone, 0.5% yeast extract, 0.1% glucose). Early stationary phase cells were harvested when a culture reached OD_600_ 1.0 (∼5 × 10^8^ cells/mL) at 18 h of incubation at 32°C in an orbital shaker. The *D. radiodurans* cells thus isolated were treated as described in the legend to Fig. 1 and irradiated acutely at 8.8 kGy/h (^60^Co), and survival was determined by colony-forming unit (CFU) assay after exposure to doses ranging from 0 to 150 kGy, with three biological replicates. Samples were serially diluted using phosphate-buffered saline (PBS) (10 mM sodium phosphate, 150 mM sodium chloride, pH 7.8), with 100 μL of dilutions plated on TGY agar plates and CFUs counted after 4–7 days at 37°C.

The *Escherichia coli* strain used was K-12 MG1655 (Daly *et al.,* 2004; Sharma *et al.,* 2017). Bacteria were inoculated into defined liquid LB medium (1% bactotryptone, 0.5% yeast extract, 1% NaCl). Early stationary phase cells were harvested when a culture reached OD_600_ 1.0 (∼5 × 10^8^ cells/mL) at 18 h of incubation at 37°C in an orbital shaker. The *E. coli* cells thus isolated were treated as described in the legend to Fig. 2B and irradiated (^60^Co), and survival was determined by CFU assay after exposure to doses ranging from 0 to 10 kGy, with three biological replicates. Samples were serially diluted using LB media and the dilutions plated on LB agar plates (100 μL per plate). CFUs were counted after 3 days at 37°C.

Spores of the WT strains of *Bacillus subtilis* (PS533), *Bacillus megaterium* (QMB1551), and *Bacillus thuringiensis* (Al Hakam) were prepared as previously described on double strength Schaeffer's glucose agar plates, which were incubated for ∼48 h at 37°C, and spores were harvested and highly purified (Nicholson and Setlow, 1990; Paidhungat *et al.,* 2000; Camilleri *et al.,* 2019). Vegetative cells of the three *Bacillus* strains were grown in LB medium, as for *E. coli* (see above). Batches of ∼1 × 10^9^ spores or vegetative cells thus isolated were treated and irradiated (^60^Co) as described in the legends to Figs. 3A, 3C, and S1A, and survival was determined by CFU assay after exposure to doses ranging from 0 to 30 kGy (^60^Co) (Ghosh *et al.,* 2011; Granger *et al.,* 2011). Samples were serially diluted in sterile PBS, 10 μL of dilutions spotted in a grid on LB agar plates, and CFUs were counted after 1–2 days at 37°C.

*Saccharomyces cerevisiae* WT strain EXF-6761 was chosen from a collection of fungi previously gauged for gamma radiation resistance. Strain EXF-6761 is a diploid and one of the most ionizing radiation–resistant yeasts reported (Sharma *et al.,* 2017). Cells were inoculated into defined liquid YPD medium (1% yeast extract, 2% peptone, 2% D-glucose). Early stationary phase cells were harvested when a culture reached OD_600_ 1.0 (∼1 × 10^8^ cells/mL) at 18 h of incubation at 32°C in an orbital shaker. Batches of ∼1 × 10^9^ vegetative cells thus isolated were treated and irradiated (^60^Co) as described in the legend to Fig. 2A, and survival was determined by CFU assay after exposure to doses ranging from 0 to 35 kGy, for three biological replicates, that is, starting with single colonies (one per replicate). Samples were serially diluted using YPD media and the dilutions plated on YPD agar plates (100 μL per plate). CFUs were counted after one week at 32°C.

### 4.2. Desiccation

Approximately 4 × 10^6^ CFU/mL vegetative bacteria and yeast cells, and *Bacillus* spores, were transferred to Costar Stripwell 96-well microplates (Corning; Catalog #9102), 25 μL per well, as described previously (Fredrickson *et al.,* 2008; Gaidamakova *et al.,* 2022). The microplates were transferred to desiccation chambers with Drierite at room temperature for 5 days, then stored in a -80°C freezer; moisture remaining in a desiccation chamber atmosphere is reduced to less than 0.005 mg/L (0.035% relative humidity). For survival monitoring following irradiation, the well contents were resuspended in 250 μL of strain-specific medium (dilution factor of 10^−1^) (PBS for all *Bacillus* samples), and the samples were serially diluted as described above. The dilutions were either plated or spotted on appropriate agar plates, which were incubated at 32°C for up to 1 week prior to determination of CFUs, all as described above.

### 4.3. EPR spectroscopy

For EPR analysis, 30 mL early stationary phase cultures (2–5 × 10^8^ cells/mL) were harvested by centrifugation, and cells were washed twice with MilliQ water (MilliQ H_2_O from “Banstead Nano Pure Diamond” [Thermo Scientific]) and resuspended in 0.3 mL 20% Glycerol (vol/vol) MilliQ water. EPR tubes (quartz, inner diameter 2.1 mm, length 65–70 mm, Wilmad-LabGlass) were filled with ∼80 μL of the concentrated cell suspensions and stored frozen at -80°C for EPR analyses.

Absorption display 35 GHz continuous-wave (CW) EPR spectra were recorded using a lab-built EPR spectrometer (Sharma *et al.,* 2017; Gaidamakova *et al.,* 2022). Absorption-display EPR spectra of frozen cells were collected in the “rapid passage” mode at 2 K as previously described (experimental conditions: MW frequency 34.9 GHz, MW power 1 mW, temperature 2 K, modulation amplitude 2 G, time constant 64 ms, scan rate 1 kG/min) (Sharma *et al.,* 2017; Gaidamakova *et al.,* 2022). Data acquisition was done by using a home-written program in LabVIEW. EPR spectra were simulated with EasySpin software (5.2.30). For details of the procedures for determining the fractional contributions of H-Mn and L-Mn to the Mn^2+^ EPR spectra, see Sharma *et al.* (2017) and Gaidamakova *et al.* (2022).

### 4.4. DNA isolation and analysis

Minipreps of *D. radiodurans* genomic DNA were performed by a protocol that employs hexadecyltrimethyl ammonium bromide (CTAB), as described previously (Daly *et al.,* 1994a). Isolation and conformational analysis of pMD66 plasmid DNA isolated from *D. radiodurans,* use of enzymatic reagents, gel electrophoresis, DNA labeling, blotting, hybridization, washing of blots, and autoradiography were as previously described; see legend to Fig. 4A and further details (Daly *et al.,* 1994b; Daly and Minton, 1995, 1996).

## Supplementary Material

Supplemental data

## Abbreviations Used

ccc: covalently closed circular
CFU: colony-forming unit
CW: continuous-wave
DPA: dipicolinic acid
DSBs: double strand breaks
EPR: electron paramagnetic resonance
GCR: galactic cosmic radiation
H-Mn: High-symmetry, small-molecule Mn^2+^ metabolite complexes
LB: Luria-Bertani
L-Mn: Low-symmetry Mn^2+^-bound enzymes and complexes
Mn-DPA: Mn-dipicolinic acid
Mn-Imi, H-Mn(Imi): imidazole-bound Mn^2+^
Mn-Pi, H-Mn(Pi): phosphate-bound Mn^2+^
MnSOD: Mn-dependent superoxide dismutase
OC: open circular
PBS: phosphate-buffered saline
ROS: reactive oxygen species
SC: supercoiled
SSBs: single strand breaks
TEM: transmission electron microscopy
TGY: tryptone-glucose-yeast extract
UVC: ultraviolet C (200–280 nm)

## Notes

Associate Editor: John Rummel
