# Supplementary material for: Effects of Desiccation and Freezing on Microbial Ionizing Radiation Survivability: Considerations for Mars Sample Return
Source: Astrobiology. 2022 Oct 31;22(11):1337–50. doi: 10.1089/ast.2022.0065 (PMC9618380; doi:10.1089/ast.2022.0065)
Supplement: Supplemental data [file Supp_FigS1-S4.docx]

**Supplemental Information for AST-2022-0065**

**Effects of Desiccation and Freezing on Microbial Ionizing Radiation Survivability: Considerations for Mars Sample-Return**

William H. Horne, Robert P. Volpe, George Korza, Sarah DePratti, Isabel H. Conze, Igor Shuryak, Tine Grebenc, Vera Y. Matrosova, Elena K. Gaidamakova, Rok Tkavc, Ajay Sharma, Cene Gostinčar, Nina Gunde-Cimerman, Brian M. Hoffman, Peter Setlow, Michael J. Daly

**Figure S1.** Gamma radiation resistance and H-Mn content of *B. thuringiensis*.

**Figure S2.** Decomposition of EPR spectrum of *B. megaterium* spores into contributions either from the two reference exemplars, H-Mn(Pi) and H-Mn(Imi) used previously, or H-Mn(Pi) and H-Mn(DPA) used in this work.

**Figure S3.** Decomposition of the H-Mn EPR spectra of endospores and vegetative cells of *B. subtilis*, *B. megaterium*, and *B. thuringiensis*.

**Figure S4.** pMD66 Plasmid. pMD66 (26,743 bp).

**Figure S1**

**
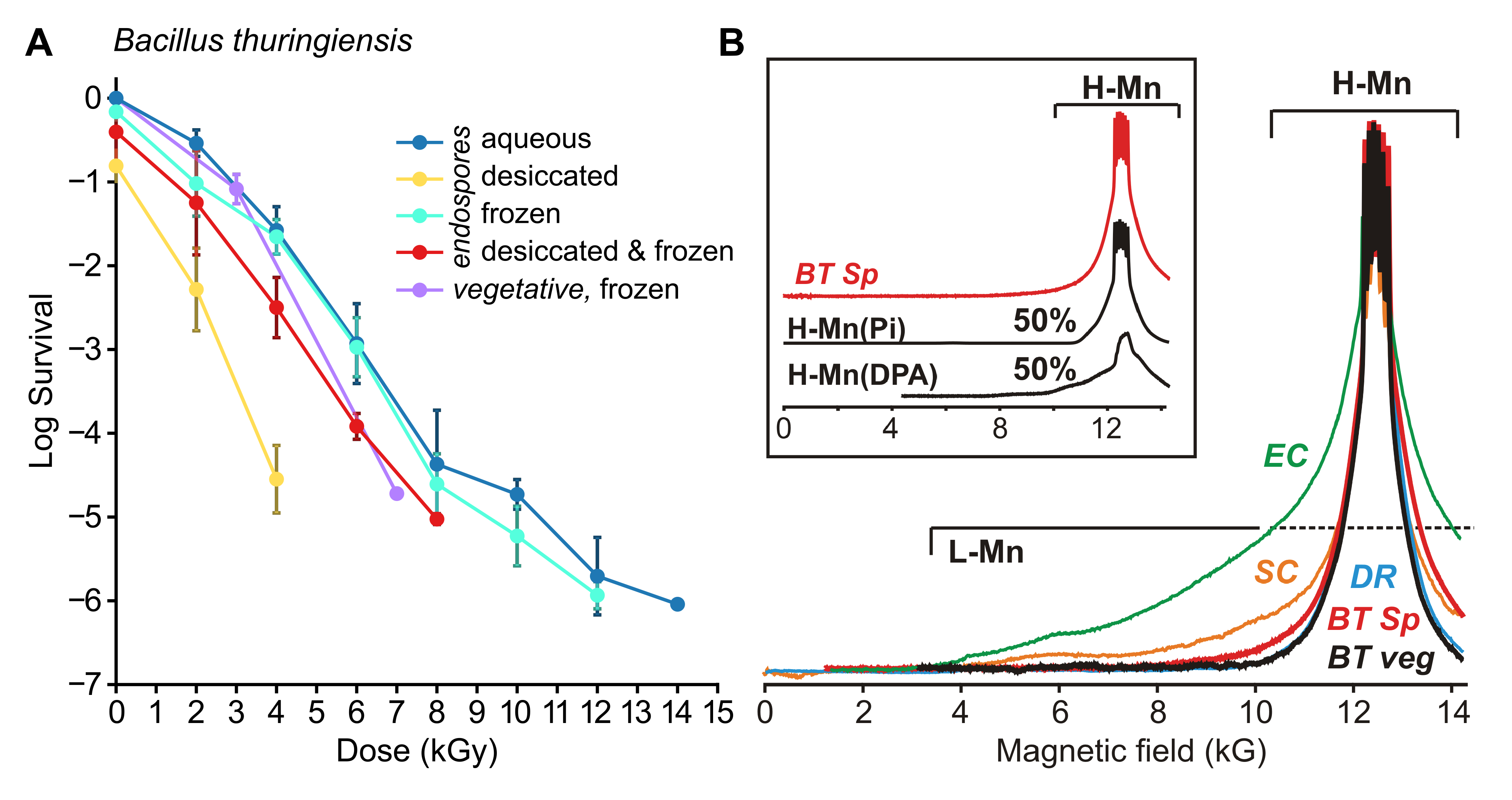
**

**Legend to Fig. S1**. Gamma radiation resistance and H-Mn content of *B. thuringiensis*. (**A**) Survival curves for *B. thuringiensis* (ATTC 10792) spores and vegetative cells. Data normalization as in Figures 1-3, main text. Following irradiation, cell survival was monitored by CFU assay, with SD error bars representing data from three experimental replicates. Data normalization as in Figure 1 (main text). (**B**) Absorption display 35 GHz continuous-wave (CW) EPR spectra were for *B. thuringiensis* spores (*BT Sp*, red) and vegetative cells (*BT veg*, black) compared to *D. radiodurans* (wild-type) (*DR*, cyan), *E. coli* (MG1655) (*EC*, green), and *S. cerevisiae* (EXF-6761) (*SC*, orange). (Inset) Decomposition of EPR spectra of *B. thuringiensis* spores (*BT Sp*) into contributions to the spectrum from the two reference exemplars, H-Mn(Pi) and H-Mn(DPA).

**Figure S2**


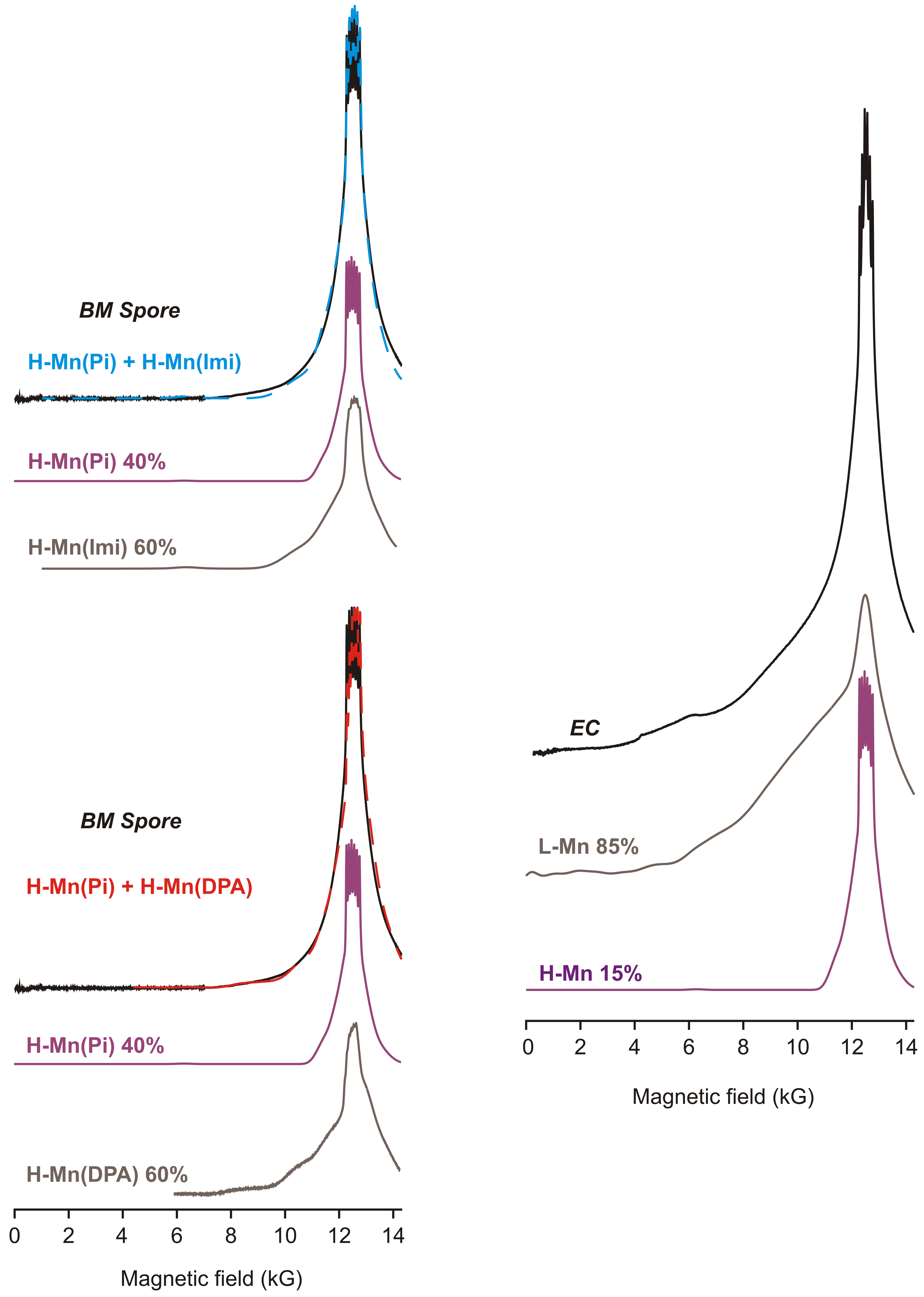


**Legend to Fig. S2.** (Left panels) Decomposition of EPR spectrum of *B. megaterium* (*BM*) spores into contributions either (upper) from the two reference exemplars, H-Mn(Pi) and H-Mn(Imi) used previously, or (lower) H-Mn(Pi) and H-Mn(DPA) used in this work. As explained in text, for the current work we have replaced the EPR spectrum of H-Mn(Imi) with H-Mn(DPA), as the two sum spectra “H-Mn(Pi)+H-Mn(Imi)” (cyan) and “H-Mn(Pi)+H-Mn(DPA)” (red), both satisfactorily match the experimental spectrum. (Right panel) Decomposition of EPR spectrum of *E. coli* (*EC*) into contributions from two reference exemplars H-Mn(Pi), and L-Mn.

**Figure S3**

**
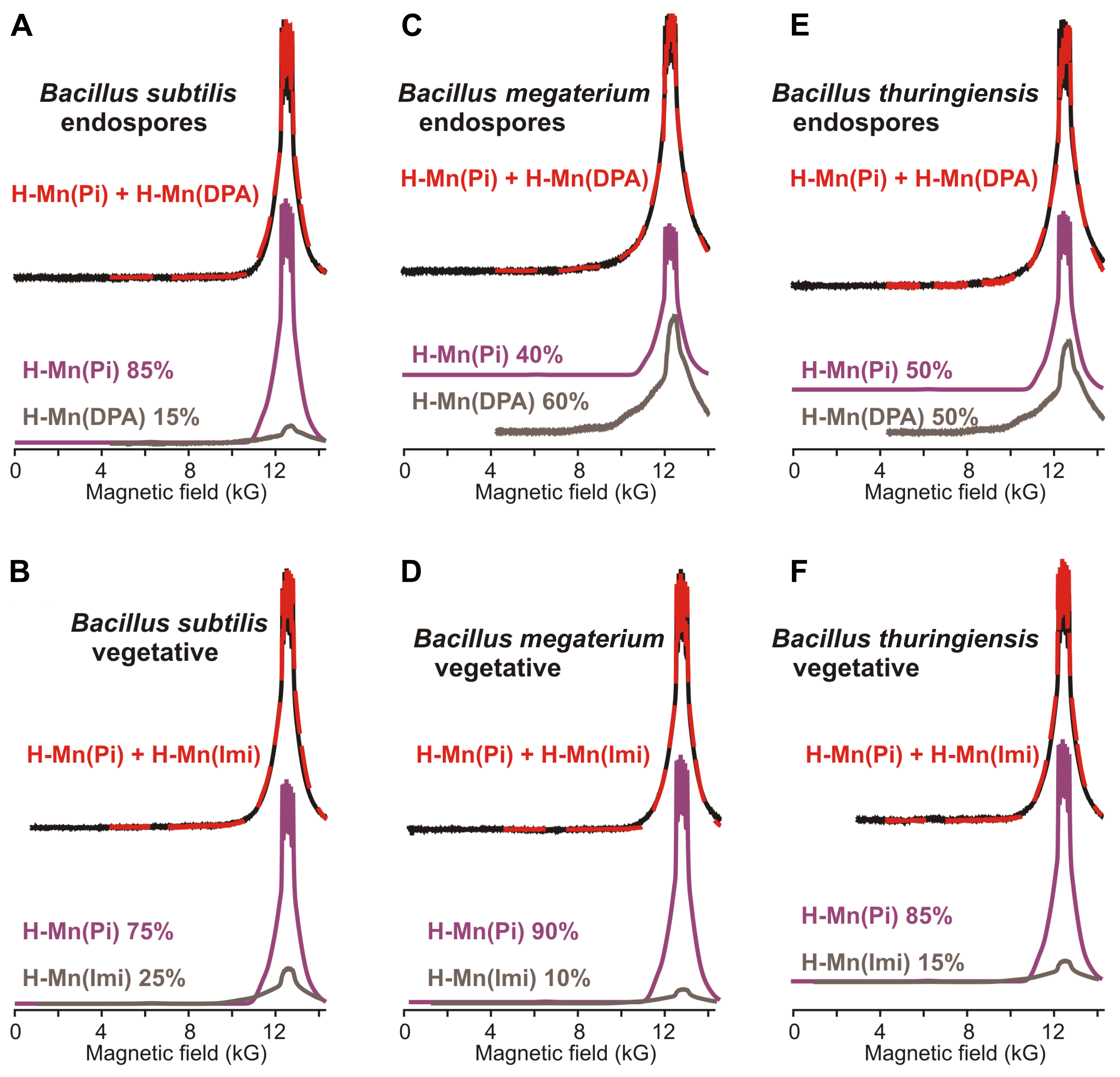
**

**Legend to Fig. S3.** As described in the main text, decomposition of the H-Mn EPR spectra of endospores and vegetative cells of *B. subtilis*, *B. megaterium*, and *B. thuringiensis* into contributions to the spectra from the two reference exemplars, H-Mn(Pi) and H-Mn(DPA) for endospores; and H-Mn(Pi) and H-Mn(Imi) for vegetative cells.

**Figure S4**

**
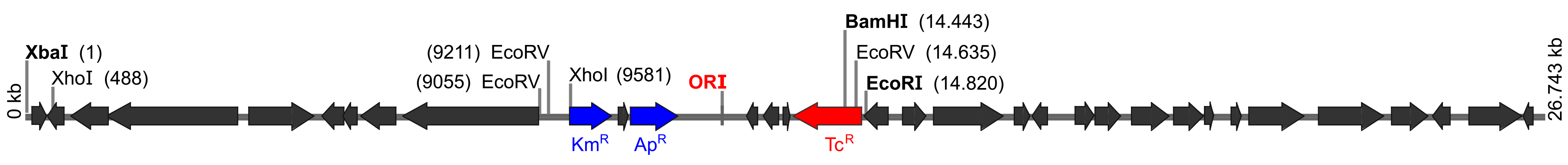
**

**Legend to Fig. S4.** pMD66 Plasmid. pMD66 (26,743 bp) is a ccc DNA plasmid derived from pUE11, a natural plasmid of *D. radiodurans* strain SARK that does not share homology with the ATCC BAA-816 *D. radiodurans* R1 genome, as reported previously (S1, S2). Molecular studies on pMD66 in WT *D. radiodurans* (strain R1, ATTC BAA-816) undergoing recovery from 17.5 kGy demonstrated that pMD66 exists at 6 copies per cell and that DSBs in pMD66 are repaired by homologous recombination mechanisms, and with the same efficiency as the R1 chromosomes (S3, S4). Analogous studies of pMD66 in the *recA*^-^ strain *rec30* showed that DSBs are not repaired when the RecA protein is inactivated; however, OC forms of pMD66 are restored to SC forms in a *recA^-^*-independent manner, as shown in Fig. 4A (main text) (S1, S2). pMD66 was chosen for the study of Fig. 4 (main text) because of its size (26.7 kbp): pMD66 exposed to 10 kGy *in vivo* suffers less than 1 DSB per ccc molecule (3.3 × 10.0 kGy × 0.0267 Mbp). This enabled us to distinguish between the three structural forms of pMD66 *in vivo* at 10 kGy, a dose at which 100% of wild-type *D. radiodurans* cells survive; and in the *D. radiodurans* *recA*^-^ strain *rec30* exposed to 10 kGy, which kills 100% of the cells (S1-S4). We thus further show that *recA*^-^ (*rec30*) *D. radiodurans* cells, which are unable to repair DSBs, succumb to ionizing radiation even though the cells hyperaccumulate H-Mn antioxidants (Fig. 3D, main text). The radiolabeled DNA probe used in the Southern blot of Fig. 4A (main text) is a unique 1.2 kb fragment of pMD66 containing the *tet* gene (Tc^R^, red) (S1, S2). The GenBank accession number for the 26,743 bp nucleotide sequence of pMD66: BankIt2569538 pMD66 ON398698.

*Note*, pMD66 is a shuttle-plasmid that replicates in *E. coli* at >50 copies per cell (S1). However, *E. coli* cells, unlike *D. radiodurans* cells, succumb to γ-rays at doses far below those needed to cause DSBs in pMD66 (Fig. 2B, main text). In fact, *E. coli* cells exposed to doses above 10 kGy suffer not only severe proteome damage, but the cells are lysed (S5-S7).

**Fig. S4 References**

S1. Daly MJ, Ouyang L, Fuchs P, Minton KW. In vivo damage and recA-dependent repair of plasmid and chromosomal DNA in the radiation-resistant bacterium Deinococcus radiodurans. J Bacteriol. 1994 Jun;176(12):3508-17. doi: 10.1128/jb.176.12.3508-3517.1994. PMID: 8206827; PMCID: PMC205538.

S2. Daly MJ, Ling O, Minton KW. Interplasmidic recombination following irradiation of the radioresistant bacterium Deinococcus radiodurans. J Bacteriol. 1994 Dec;176(24):7506-15. doi: 10.1128/jb.176.24.7506-7515.1994. PMID: 8002574; PMCID: PMC197207.

S3. Daly MJ, Minton KW. Interchromosomal recombination in the extremely radioresistant bacterium Deinococcus radiodurans. J Bacteriol. 1995 Oct;177(19):5495-505. doi: 10.1128/jb.177.19.5495-5505.1995. PMID: 7559335; PMCID: PMC177357.

S4. Daly MJ, Minton KW. An alternative pathway of recombination of chromosomal fragments precedes recA-dependent recombination in the radioresistant bacterium Deinococcus radiodurans. J Bacteriol. 1996 Aug;178(15):4461-71. doi: 10.1128/jb.178.15.4461-4471.1996. PMID: 8755873; PMCID: PMC178212.

S5. Gaidamakova EK, Myles IA, McDaniel DP, Fowler CJ, Valdez PA, Naik S, Gayen M, Gupta P, Sharma A, Glass PJ, Maheshwari RK, Datta SK, Daly MJ. Preserving immunogenicity of lethally irradiated viral and bacterial vaccine epitopes using a radio- protective Mn2+-Peptide complex from Deinococcus. Cell Host Microbe. 2012 Jul 19;12(1):117-124. doi: 10.1016/j.chom.2012.05.011. PMID: 22817993; PMCID: PMC4073300.

S6. Sharma A, Gaidamakova EK, Matrosova VY, Bennett B, Daly MJ, Hoffman BM. Responses of Mn2+ speciation in Deinococcus radiodurans and Escherichia coli to γ-radiation by advanced paramagnetic resonance methods. Proc Natl Acad Sci U S A. 2013 Apr 9;110(15):5945-50. doi: 10.1073/pnas.1303376110. Epub 2013 Mar 27. PMID: 23536297; PMCID: PMC3625348.

S7. Sharma A, Gaidamakova EK, Grichenko O, Matrosova VY, Hoeke V, Klimenkova P, Conze IH, Volpe RP, Tkavc R, Gostinčar C, Gunde-Cimerman N, DiRuggiero J, Shuryak I, Ozarowski A, Hoffman BM, Daly MJ. Across the tree of life, radiation resistance is governed by antioxidant Mn^2+^, gauged by paramagnetic resonance. Proc Natl Acad Sci U S A. 2017 Oct 31;114(44):E9253-E9260. doi: 10.1073/pnas.1713608114. Epub 2017 Oct 17. PMID: 29042516; PMCID: PMC5676931.
